# Support for age related firearm policies among Black American firearm owners: examining the role of children in the home

**DOI:** 10.1186/s12887-025-05781-8

**Published:** 2025-06-05

**Authors:** Vanya C. Jones, Elizabeth D. Wagner, Julie A. Ward, Rebecca Valek, Cassandra Crifasi

**Affiliations:** 1 Johns Hopkins Bloomberg School of Public Health, 615 N Wolfe Street, Baltimore, MD 21205 USA; 2 Department of Health Policy and Management, Johns Hopkins Bloomberg School of Public Health, 750 E Pratt Street, 15th floor, Baltimore, MD 21202 USA; 3 Department of Medicine, Health, and Society, Vanderbilt College of Arts and Science, 2301 Vanderbilt Place, Nashville, TN 37235 USA; 4 Oregon Health & Science, University-Portland State University School of Public Health, 1810 SW 5th Ave, Portland, OR 97201 USA

**Keywords:** Firearm owner, Firearm policy, Children in the home

## Abstract

**Background:**

In recent years, ownership of firearms has increased to approximately one-third of American households. Firearm access is a threat to child safety, with the most common location of fatal childhood firearm injury being at home. Exploring differences in opinions on gun violence prevention policies among Black firearm owners could inform community-supported interventions as one approach to reduce violent injuries among disproportionately affected youth. This study examined support for child-safety-related firearm policies among Black gun owners and whether support differed between those with and without children living in the home.

**Methods:**

This observational cross-sectional study used data from the 2023 wave of the National Survey of Gun Policy, fielded using NORC’s AmeriSpeak Panel. Black survey respondents (*n* = 177) self-reported firearm ownership and whether children lived in the home. Fourteen key policies potentially relevant to youth firearm violence, firearm exposure, and safety were evaluated in this study using a binary measure of support derived from a five-point Likert scale. Survey weights were applied to adjust for sampling deviations, and survey-weighted proportions (WPs) and 95% confidence intervals (CIs) were calculated to examine differences between groups.

**Results:**

Black firearm owners with children in the home (*n* = 65) were younger, had lower income, and were more likely to live in metropolitan areas compared to those without children in the home (*n* = 112). There was over 60% support, with little variance, among Black firearm owners with and without children living in the home for 11 of the 14 policies. The only significant difference among these groups was in support for temporary firearm removal by law enforcement for individuals posing an immediate threat. Although still over 60%, support was lower among owners with children living in the home (65%, CI: 0.51–0.79) compared to those without children living in the home (85%, CI: 0.77–0.93).

**Conclusions:**

This study found that Black firearm owners are a demographically diverse group overall and when comparing those with and without children living in the home. These findings suggest that Black firearm owners may be receptive to policies and programs to reduce violence among pediatric populations.

**Supplementary Information:**

The online version contains supplementary material available at 10.1186/s12887-025-05781-8.

## Background

Firearm ownership has increased across all demographic groups in recent years, including among those with historically lower rates of ownership [1–3]. According to the Pew Foundation, in.

2023, approximately one-third of Americans own firearms [4]. Perceptions of firearm ownership vary significantly within communities disproportionately affected by firearm violence; some individuals see firearm possession as a protective measure, while others perceive it as heightening the risk of harm [5]. However, consistently across groups, self-defense or protection against other people is reported as the major reason for firearm ownership [6, 7], including homes with children [8, 9]. This is in direct conflict with the prevailing epidemiology that firearm exposure increases the risk of homicide and suicide in the home [10].

Firearm violence is a critical health challenge in the United States, particularly among children, teens, and emerging adults, for whom firearm injury is the leading cause of death [11, 12]. Public health interventions to reduce firearm violence should focus on strategies that are both evidence based and grounded in the needs of the most-affected communities [13]. For example, Black youth living in urban neighborhoods experience disproportionate exposure to firearm violence, both through direct involvement and indirectly through community-level exposure [14–16]. These disparities are shaped by structural inequities such as historical and contemporary racial segregation, economic disinvestment, and systemic underfunding of schools, housing, and other essential resources [17–19]. Furthermore, school safety policies have been identified as shaping educational and health related disparities for Black youth [20, 21]. For example, policies such as the Gun-Free Schools Act of 1994, the catalyst for the current zero tolerance policies for schools, have been used to increase youth arrests in schools, disproportionately impacting Black students [22, 23]. Support for firearm policies among parents may be influenced by such experiences with historic policies, as well as by the disproportionate burden of firearm injury among youth, particularly Black youth.

The most common location of fatal firearm injury among children under 17 is in the home, regardless of injury intent, and injuries intentionally inflicted by youth frequently involve firearms obtained from their homes [24, 25]. Families with firearms in the home may help identify effective and preferred strategies to reduce youth violence. Interventions that mitigate firearm owners’ concerns are critical in fostering safety for the owner and their family, while balancing overall well-being for children.

Research has explored the reasons for firearm ownership and the levels of support for firearm related policies across diverse demographic groups. Findings suggest that, while support for protective policies is generally high, some differences in opinions on firearm policies exist based on sex, race and ethnicity, and geographic location [26–29]. While previous research has found high support for firearm policies among Black Americans, questions remain as to whether having children in the home changes support [26]. Highlighting that less is known about within-group differences in opinions on gun violence prevention policies among firearm ownerships, particularly among Black communities disproportionately affected by youth violence or violence against youth. Understanding differences could help inform community-supported interventions. Using public opinion data from the National Survey of Gun Policy, this study describes the demographic characteristics of Black firearm owners with and without children living in the home. This exploratory investigation also examines the support for various firearm-related policies among Black firearm owners, focusing on whether support differed between those with and without children living in the home.

## Methods

In this observational cross-sectional study, data from the 2023 wave of the National Survey of Gun Policy were used. The survey was fielded from January 4 to February 6, 2023, using NORC at the University of Chicago’s AmeriSpeak Panel, a nationally representative, probability-based sample of U.S. adults [30]. The AmeriSpeak Panel was generated from the U.S. Postal Service Delivery Sequence File with supplemental field surveillance efforts to improve coverage of rural communities, low-income residents, and those who identify politically as conservative [31]. The National Survey of Gun Policy, conducted online and over the phone, included both English and Spanish-speaking participants, with an oversample of Black Americans [3]. The overall completion rate for the survey was 77% (*n* = 3096) [3, 31]. Our analysis focused on respondents who self-identified as Black and firearm-owning (*n* = 177 of 665 Black respondents). Respondents answered demographic questions about their sex, age, education, employment, marital status, and political party affiliation, as well as household composition. The number of children in the home was reported using four age-specific categories (0–1, 2–5, 6–12, and 13–17), and adults were defined as individuals aged 18 and older. Consistent with our analytic aim, we collapsed all child age categories (< 18 years) into a dichotomous indicator for the presence of at least one child in the home (yes/no). Firearm ownership was determined based on self-reported affirmative responses to two questions: “Do you happen to have in your home or garage any guns or revolvers?” and “Do any of these guns personally belong to you?”.

Policy-related items potentially relevant to youth firearm violence, firearm exposure, and safety were evaluated in this study. This study examined approaches included policies related to prohibited persons, safe storage, public carry, funding, and temporary firearm removal (see the Supplemental Appendix 1 for the specific survey questions). Survey respondents were asked to rank their approval of the 14 examined policies on a five-point Likert scale, with options ranging from “strongly favor” to “strongly oppose.” To estimate the support for each policy, binary categorical variables were created comparing support to neutral and non-support. Analysis was on completed survey responses, if a respondent did not answer a question, they were excluded from that analysis. The analysis was conducted using survey weights provided by NORC to adjust for known sampling deviations, oversampling, and survey nonresponse to ensure the sample was representative of the U.S. population. Using these weights, a survey design object was created with the survey function in R (version 4.0.2). First, survey-weighted proportions (WPs) with 95% confidence intervals (CIs) were calculated for demographic characteristics and for each policy item, stratified by having at least one child in the home or not. Any 95% CI whose lower bound fell below zero due to limited sample sizes was truncated at zero and annotated in the tables. Next, for each policy item, we fit a separate simple logistic regression using the svyglm function to compare policy support between owners with and without children in the home. Effect sizes are presented as odds ratios (ORs) with 95% CIs, and results discussed in the text are limited to findings that are significant based on an alpha value of < 0.05.

A complete list of OR’s is provided in Appendix Table 2.

## Results

### Demographics

Of the 177 Black firearm owners, 37% (*n* = 65) reported having at least one child living in the home. The number of children reported per household ranged from one to six: 1 child in the home = 30%, 2 = 37%, 3 = 14%, 4 = 12%, 5 = 5%, 6 = 1%. Overall, respondents reported a total of 105 children. Within the child age specific categories, there were 9 children reported as aged 0–1, 21 aged 2–5, 41 aged 6–12, and 34 aged 13–17 (representing 9%, 20%, 39%, and 32% of all reported children, respectively).

In a comparison of Black firearm owners with children living in the home to those without, respondents differed across several demographic characteristics (Table 1). Income levels among those with children living in the home were lower, with 48% earning below $35,000 (CI: 0.32– 0.64) compared to 33% without children (CI: 0.20–0.45). Age distributions varied: 46% of those with children living in the home were aged 18–34 (CI: 0.30–0.62), while 74% of those without were aged 50 or older (CI: 0.50–0.98).

**Table 1:** Firearm ownership among black Americans by household composition

Variable	Subgroup	Black Firearm Owner *n* = 17 % (CI) *	Black Firearm Owner & Has Children *n* = 65 %(CI) ^*^	Black Firearm Owner & No Children *n* = 112 % (CI) ^*^
**Income**	>$75,000	31 (22, 39)	31 (17, 44)	31 (20, 42)
	$35,000-$74,999	31 (22, 39)	21 (11, 32)	36 (25, 48)
	<$35,000	38 (29, 48)	48 (32, 64)	33 (20, 45)
**Age**	> 65	27 (19, 36)	8 (1, 14)	39 (27, 51)
	50–64	29 (20, 38)	19 (8, 31)	35 (23, 47)
	35–49	21 (14, 0.28)	27 (15, 39)	17 (8, 25)
	18–34	23 (14, 32)	46 (30, 62)	9 (1, 18)
**Sex**	Male	56 (47, 66)	46 (30, 62)	62 (50, 04)
	Female	44 (34, 53)	54 (38, 70)	38 (26, 50)
**Educa0on**	High school graduate or less	47 (37, 56)	40 (24, 56)	50 (38, 63)
	Some college	28 (20, 36)	35 (20, 50)	24 (15, 33)
	Bachelor’s or higher	25 (19, 32)	25 (14, 35)	26 (17, 34)
**Employment**	Employed	57 (47, 66)	64 (49, 79)	52 (40, 65)
	Not employed	7 (2, 12)	15 (3, 27)	2 (0, 5) ^^^
	Not working - another reason	36 (27, 45)	21 (9, 33)	45 (33, 58)
**Children in the Home**	No	63 (53, 72)	-	-
	Yes	37 (28, 47)	-	-
**Adults in the Home**	Only one adult in the home	32 (23, 41)	30 (17, 43)	33 (22, 45)
	More than one adult in the home	68 (59, 77)	70 (57, 83)	67 (55, 78)
**Urbanicity**	Metro area	86 (80, 93)	93 (87, 99)	82 (72, 92)
	Non-metro area	14 (7, 20)	7 (1, 13)	18 (8, 28)
**Region**	Midwest	13 (8, 19)	13 (2, 23)	14 (8, 20)
	Northeast	8 (2, 14)	6 (0,11) ^^^	9 (00, 18)
	South	75 (67, 83)	79 (67, 91)	72 (62, 83)
	West	4 (1, 7)	3 (1, 7)	5 (0, 9)
**Marital Status**	Married	49 (39, 59)	54 (39, 69)	46 (34, 58)
	Never married	29 (20, 38)	29 (15, 43)	28 (17, 40)
	Widowed/Divorced	22 (15, 30)	17 (8, 26)	26 (15, 36)
**Party ID**	Democrat	65 (56, 75)	49 (34, 65)	75 (66, 85)
	Independent	32 (23, 41)	48 (32, 64)	0.22 (13, 31)
	Republican	3 (1, 5)	3 (0, 6) ^^^	3 (0, 6) ^^^

^*^Weighted proportions were estimated using the survey package in R

^^^Due to low cell counts, some estimated proportion CI were capped at zero

There were some differences in sex among Black gun owners as well, with 54% of respondents with children living in the home being female (CI: 0.38–0.70), compared to 38% of those without children in the home (CI: 0.26-0.50). Education levels were similar, but slightly fewer firearm owners with children living in the home had a high school diploma or less (40%, CI: 0.24–0.56), compared to those without children living in the home (50%, CI: 0.38–0.63). Employment status also differed: 64% of firearm owners with children living in the home were employed (CI: 0.49– 0.79) compared to 52% without (CI: 0.40–0.65). 45% of firearm owners without children in the home reported not working for other reasons, such as retirement or temporary layoff (CI: 0.33–0.58).

Most Black firearm owners lived in metropolitan areas, but this was more common among respondents with children living in the home (93%, CI: 0.87–0.99) than those without (82%, CI: 0.72–0.92). Regionally, both groups were concentrated in the South, with 79% of those with children living in the home (CI: 0.67–0.91) and 72% of those without (CI: 0.62–0.83) residing in the region. Marital status showed modest differences: 54% of firearm owners with children living in the home were married (CI: 0.39–0.69), compared to 46% without (CI: 0.34-0.58). Party identification differed more significantly, with 49% of firearm owners with children in the home identifying as Democrats (CI: 0.34–0.65) and 48% as Independents (CI: 0.32–0.64). Among those without children in the home, 75% identified as Democrats (CI: 0.66–0.85) and 22% as.

Independents (CI: 0.13–0.31). Republican affiliation was reported by 3% of both groups (see Table 1).

### Policy support

The variance in support for most firearm policies between Black firearm owners with and without children living in the home was not statistically significant. Both groups reported over 60% support for 11 of the 14 policies (see Table 2). 72% of firearm owners with children living in the home expressed support for banning handgun ownership for individuals under 21 (CI: 0.60–0.84) compared to 82% of those without (CI: 0.72–0.93). Support for secure firearm storage was nearly identical between groups, with 74% of owners with children living in the home (CI: 0.61–0.87) and 75% of those without (CI: 0.63–0.86) favoring the policy. 70% of firearm owners with children living in the home supported dispatching clinicians to accompany police on mental health calls (CI: 0.57–0.83) compared to 82% of those without (CI: 0.72–0.92). Community-based mental health programs garnered over 80% support from both groups (children in the home: 84%, CI: 0.73–0.96; no children in the home: 82%, CI: 0.72–0.91). Family-initiated, court-ordered firearm removal was also widely supported, with 74% support among those with children living in the home (CI: 0.61–0.87) versus 83% for those without (CI:

0.72–0.94). There was one notable difference regarding support for firearm access restrictions: Owners with children living in the home were less likely to support temporary firearm removal by law enforcement for individuals posing an immediate threat (65%, CI: 0.51–0.79) compared to those without children (85%, CI: 0.77–0.93). This difference was also statistically significant via simple logistic regression. Owners with children had 68% lower odds of supporting the policy compared to those without children (OR = 0.32, CI: 0.13–0.79, *p* = 0.014).

**Table 2:** Support for child-safety related firearm policies among black gun owners by household composition

Policies	Black Firearm Owner and Has Children in Home % (CI)*	Black Firearm Owner and No Children in Home %(CI)*
**Prohibited persons policies**		
Prohibiting a person under the age of 21 from having a handgun	72 (60, 84)	82 (72, 93)
Prohibiting a person subject to a temporary domestic violence restraining order from having a firearm for the duration of the order	86 (77, 94)	78 (66, 89)
Extending domestic violence-related firearm prohibitions to include couples who have dated	66 (52, 81)	62 (49, 74)
Prohibiting a person convicted of two or more DWI or DUIs in a five-year period from having a firearm for five years	69 (56, 82)	57 (45, 70)
Prohibiting a person convicted of two or more misdemeanor crimes involving illegal drugs in a five-year period from having a firearm for five years	62 (48, 76)	65 (53, 76)
**Safe storage policies**		
Requiring by law that a person lock up the firearms in their home when not in use to prevent handling by children or teenagers without adult supervision	74 (61, 87)	75 (63, 86)
**Policies on carrying guns in public**		
Allowing a person who can legally carry a concealed firearm to bring that firearm onto school grounds for kindergarten through 12th grade	34 (19, 50)	22 (11, 33)
**Funding-related policies**		
Funding community-based firearm violence prevention programs that provide outreach, conflict mediation, and social support for individuals at high risk of firearm violence	81 (71, 90)	83 (73, 92)
Directing public funding to dispatching a clinician to accompany police officers on calls involving individuals displaying symptoms of mental illness	70 (57, 83)	82 (72, 92)
Directing public funding for community-based mental health programs to respond to calls involving individuals displaying symptoms of mental illness	84 (73, 96)	82 (72, 91)
Redirecting government funding currently spent on the police to social services for people at risk of firearm violence	65 (52, 79)	50 (38, 63)
Funding, through public insurance, hospital-based arm violence prevention programs that offer counseling to address psychological trauma	76 (65, 88)	76 (65, 88)
**Temporary firearm removal policies**		
Authorizing law enforcement officers to temporarily remove firearms from individuals who the officer determines pose an immediate threat of harm to self or others	65 (51, 79)	85^+^ (77, 93)
Allowing family members to ask the court to temporarily remove firearms from a relative who they believe was at risk of harming himself or others	74 (61, 87)	83 (72, 94)

*Weighted proportions were estimated using the survey package in R. Sample sizes varied due to exclusion of missing values per policy question

^+^Indicates a statistically significant (*p* < 0.05) relationship with policy support from simple logistic regression

Policies that received lower than 60% support from either group of Black firearm owners included redirecting police funding to social services (children in the home: 65%, CI: 0.52–0.79; no children in the home: 50%, CI: 0.38–0.63) and prohibiting firearm ownership for individuals with multiple citations for driving under the influence of alcohol or other substances (DUIs) (children in the home: 69%, CI: 0.56–0.82; no children in the home: 57%, CI: 0.45–0.70). For both groups, support was low for policies allowing concealed carry-on school grounds, with only 34% (CI: 0.19–0.50) of Black firearm owners with children living in the home and 22% (CI: 0.11–0.33) of those without favoring the measure (see Table 2).

## Discussion

This study found that Black firearm owners are a demographically diverse group overall and when comparing those with and without children living in the home. Black firearm owners with children living in the home tended to have lower income, be younger in age, and live in metropolitan areas, supporting other findings about overall firearm owners with children in the US [32. There was a notable difference in political affiliation; those with children in the home were less likely to identify as a Democrat and more likely to identify as Independent. Political affiliation has been found to be correlated with differences in reasons for firearm ownership, support for firearm policies, and firearm storage practices [29]. Future research should explore whether these differences by political affiliation support the need for targeted firearm safety messages to support responsible practices, especially as firearm owners continue to diversify. Recent research described an increase in Black American firearm owners overall and identified that protection was a primary factor for this change [1, 32]. Safety concerns motivating firearm purchasing among Black firearm owners may include protection from community violence, racial violence, political violence, and protection against police violence [3, 31, 33, 34]. Concerns about safety may be driven by consistently high rates of homicides and neighborhood crime, the increase of hate crimes targeting ethnic and racial groups, and recent US political unrest [3, 31,34, 35]. In this context, our findings of high support for funding community violence interventions and community-based mental health services may indicate a perceived responsiveness of these approaches to conditions that may have also motivated gun ownership. In our study, a larger proportion of Black firearm owners with children living in the home identified as female compared to those without. While this cross-sectional study cannot identify causality, prior research provides insights into what may motivate gun ownership among women with children living in the home. Related to perceptions of safety, fear for another person, also known as altruistic fear, combined with the concern of individual vulnerability may shed light on differences in firearm ownership among women with children living in the home compared to those without [36]. Prior research has found that children living in the home has been linked to an increase in firearm ownership for women who are experiencing financial distress during times of economic instability [37]. Expanding on this, the felt need to protect children from police violence, neighborhood crime, and hate crimes may create a multifaceted rationale for Black women with children living in their homes to have a firearm as a measure of control and safety.

 [38]. Future research should further explore such motivations among firearm owners, especially Black firearm owners with children in the home, to better understand the interplay between demographics, perceptions of safety, and firearm ownership. This may also represent an opportunity to engage with Pediatricians and other health professionals who work with children and families to provide high-quality safe and secure firearm storage counseling should parents choose to have firearms in the home [39].

While there were some differences in demographic characteristics among firearm ownership with and without children living, these findings are consistent with prior research finding that firearm owning Black Americans tend to favor more restrictive gun policies and efforts to expand community-based gun violence prevention [26]. In our current study, there was high support for most of the policies assessed; eleven out of the fourteen policies examined had greater than 60% support from both groups regardless of whether there were children in the home. These policies included higher support for explicitly age-related policies such as prohibiting a person under the age of 21 from having a handgun and requiring by law that a person lock up the firearms in their home when not in use to prevent handling by children or teenagers without adult supervision. The exception to these findings was the level of policy support for authorizing law enforcement officers to temporarily remove firearms from individuals who the officer determines pose an immediate threat of harm to self or others. While this policy had over 60% support from both groups, firearm owners without children in the home expressed greater support. One reason for this difference could be the concern of protecting children from violence, including violence by police, as the policy requires law enforcement involvement.

This study provides a unique contribution to our understanding of the diversity of firearm ownership; however, there are some limitations to consider when interpreting these results. This analysis could be affected by over- or under- sampling of specific groups or differential response rates among subpopulations, which may have introduced bias. To mitigate this, survey weights to account for sampling deviations and differences in response rates were used to adjust the sample, so that the data reflected the demographic composition of Black adults in the U.S. Small sample sizes in some of the subgroups may have reduced the precision, power, and stability of some estimates, which can be seen in the CI’s for certain estimates of the WPs for demographic variables that contained negative numbers but were capped at zero (Table 1). This did not appear to be an issue for WPs of policy support. There may have been differences in support by characteristics of respondent or their home, including age of child(ren) or number of children living in the home or number of adults in the home; due to the small sample, we were unable to explore those differences in the study. There could be under reporting of firearm ownership and children in the home. Lastly, missing data, characterized as non-response on demographic and policy survey questions, could have influenced these findings. Scattered missing responses across the fourteen policies led to varying sample sizes for each policy analysis, limiting direct comparability. To maintain statistical power, each policy analysis was conducted using the available sample. While this approach limits comparability between policies, it aligns with the study’s descriptive focus. This study does report on gun type or individual experiences with gun violence, limiting the generalizability of the findings. The limitations of this study underscore the importance of learning more about Black American firearm owners and their perceptions and practices to reduce firearm injuries and deaths.

## Conclusions

Firearm injuries and death are a leading public health crisis that has a disproportionate effect on Black Americans, particularly Black youth. Regardless of whether there were children in the home, Black firearm owners were broadly supportive of numerous policies that could impact exposure to firearms among children and teens. Pediatricians and other healthcare professionals are often credible messengers for a variety of safety topics. These findings suggest that Black firearm owners may be receptive to policies and programs to reduce violence among pediatric populations.

## Electronic supplementary material

Below is the link to the electronic supplementary material.

Supplementary Material 1

## Data Availability

This study uses the NORC the National Survey of Gun Policy. The datasets generated and/or analyzed during the current study are not publicly available as analyses are ongoing, but the datasets will be made available to qualified researchers subject to the terms of a data use agreement.

## Abbreviations

NORC: National Opinion Research Center
WPs: Weighted proportions (WPs)
Cis: Confidence intervals
