# Supplementary material for: Support for age related firearm policies among Black American firearm owners: examining the role of children in the home
Source: BMC Pediatr. 2025 Jun 5;25:459. doi: 10.1186/s12887-025-05781-8 (PMC12139284; doi:10.1186/s12887-025-05781-8)
Supplement: Supplementary file 1 — Supplementary Material 1 [file 12887_2025_5781_MOESM1_ESM.pdf]

Appendix

Table 1. Child-safety related firearm policies survey items and responses						
**Policies**						
**Prohibited persons policies**		STRONGLY FAVOR<br>1	SOMEWHAT FAVOR<br>2	NEITHER FAVOR NOR OPPOSE<br>3	SOMEWHAT OPPOSE<br>4	STRONGLY OPPOSE<br>5
1	Prohibiting a person under the age of 21 from having a handgun					
2	Prohibiting a person subject to a temporary domestic violence restraining order from having a firearm for the duration of the order					
3	Extending domestic violence-related firearm prohibitions to include couples who have dated					
4	Prohibiting a person convicted of two or more DWI or DUIs in a five-year period from having a firearm for five years					
5	Prohibiting a person convicted of two or more misdemeanor crimes involving illegal drugs in a five-year period from having a firearm for five years					
**Safe storage policies**						
6	Requiring by law that a person lock up the firearms in their home when not in use to prevent handling by children or teenagers without adult supervision					
**Policies on carrying guns in public**						
7	Allowing a person who can legally carry a concealed firearm to bring that firearm onto school grounds for kindergarten through 12th grade					
**Funding-related policies**						
8	Funding community-based firearm violence prevention programs that provide outreach, conflict					

	mediation, and social support for individuals at high risk of firearm violence					
9	Directing public funding to dispatching a clinician to accompany police officers on calls involving individuals displaying symptoms of mental illness					
10	Directing public funding for community-based mental health programs to respond to calls involving individuals displaying symptoms of mental illness					
11	Redirecting government funding currently spent on the police to social services for people at risk of firearm violence					
12	Funding, through public insurance, hospital-based firearm violence prevention programs that offer counseling to address psychological trauma					
**Temporary firearm removal policies**						
13	Authorizing law enforcement officers to temporarily remove firearms from individuals who the officer determines pose an immediate threat of harm to self or others					
14	Allowing family members to ask the court to temporarily remove firearms from a relative who they believe was at risk of harming himself or others					

Table 2. Odds Ratios of support for child-safety related firearm policies among Black gun owners by household composition

Policies	OR*	95 CI%
**Prohibited persons policies**		
Prohibiting a person under the age of 21 from having a handgun	.55	.21, 1.43
Prohibiting a person subject to a temporary domestic violence restraining order from having a firearm for the duration of the order	1.72	.65, 4.50
Extending domestic violence-related firearm prohibitions to include couples who have dated	1.21	.52, 2.80
Prohibiting a person convicted of two or more DWI or DUIs in a five-year period from having a firearm for five years	1.65	.75,3.64
Prohibiting a person convicted of two or more misdemeanor crimes involving illegal drugs in a five-year period from having a firearm for five years	.89	.41, 1.96
**Safe storage policies**		
Requiring by law that a person lock up the firearms in their home when not in use to prevent handling by children or teenagers without adult supervision	.99	.40, 2.47
**Policies on carrying guns in public**		
Allowing a person who can legally carry a concealed firearm to bring that firearm onto school grounds for kindergarten through 12th grade	1.84	.72, 4.68
**Funding-related policies**		
Funding community-based firearm violence prevention programs that provide outreach, conflict mediation, and social support for individuals at high risk of firearm violence	.85	.34, 2.15
Directing public funding to dispatching a clinician to accompany police officers on calls involving individuals displaying symptoms of mental illness	.52	0.21, 1.3
Directing public funding for community-based mental health programs to respond to calls involving individuals displaying symptoms of mental illness	1.19	.40, 3.53
Redirecting government funding currently spent on the police to social services for people at risk of firearm violence	1.85	.84, 4.07
Funding, through public insurance, hospital-based firearm violence prevention programs that offer counseling to address psychological trauma	1	.41, 2.45
**Temporary firearm removal policies**		
Authorizing law enforcement officers to temporarily remove firearms from individuals who the officer determines pose an immediate threat of harm to self or others	.32	.13, .79^{+}
Allowing family members to ask the court to temporarily remove firearms from a relative who they believe was at risk of harming himself or others	.60	.21, 1.68

*Survey weighted- ORs were obtained using simple logistic regression with the `svyglm` function in R. Sample sizes varied due to exclusion of missing values per policy question.
$^{+}$Indicates a statistically significant ($p < 0.05$) relationship with policy support from simple logistic regression.
